# Bilateral single-staged arthroscopic rotator cuff repair is comparable to staged procedures: a retrospective follow up study of 2 years

**DOI:** 10.1186/s12891-021-04304-7

**Published:** 2021-05-04

**Authors:** Chen Wang, Pu Yang, Dongfang Zhang, In-Ho Jeon, Tengbo Yu, Yingze Zhang, Chao Qi

**Affiliations:** 1 Department of Sports Medicine, the Affiliated Hospital of Qingdao University, Shandong 266103 Qingdao, P.R. China; 2 Department of Orthopedic Surgery, College of Medicine, Asan Medical Center, University of Ulsan, 88, Olympic-ro 43-gil, Songpa-gu, 05505 Seoul, Republic of Korea

**Keywords:** Rotator cuff repair, Arthroscopy, Clinical outcomes, Single-stage surgery, Staged surgery

## Abstract

**Background:**

Bilateral rotator cuff tears are not uncommon and the timing of the surgical treatment of both shoulders is debated. In the present study, we aimed to compare the clinical outcomes of patients who underwent single-stage or staged bilateral arthroscopic rotator cuff repair.

**Methods:**

From March 2013 to May 2018, a retrospective review on all patients who underwent bilateral arthroscopic rotator cuff repair at our department was performed. Patients were separated into 2 groups: single-stage and staged. The minimum follow-up period was 2 years. The visual analog scale (VAS), American Shoulder and Elbow Surgeons (ASES) score, University of California, Los Angeles (UCLA) score, Constant-Murley (Constant) score, the range of motion (ROM) of the shoulder and the hospitalization costs were evaluated for comparison between the two groups before and after the operation. Differences between groups were assessed using t-tests and ANOVA.

**Results:**

All 51 patients completed follow-up of 2 years, single stage (*n* = 24) and staged group (*n* = 27). There was no significant difference in the VAS, ASES, UCLA and Constant scores between the single-stage group and the staged group before the operation. Postoperative clinical scores were significantly improved in both groups (*P* < 0.05). All outcome scores were significantly different between the two groups at 6 months postoperatively, and the staged scored better than the single-stage (*P* < 0.05). At 12, 18, and 24 months after the operation, the outcome scores were not significantly different between the two groups. At follow-up, the ROM of the shoulder was not significantly different between the two groups. In the single-stage group, the outcome scores and ROM were similar for both shoulders and comparable to the staged group. We also found significant cost savings in the single-stage group (4440.89 ± 130.55 USD) compared to the staged group (5065.73 ± 254.76 USD) (*p* < 0.05).

**Conclusions:**

Patients receiving single-stage or staged bilateral arthroscopic rotator cuff repair showed similarly good clinical outcomes at follow-ups longer than 6 months. Moreover, good outcomes were observed on both sides of the single-stage group.

## Background

With a growing number of patients suffering from rotator cuff tears, surgical repair is the established treatment for full thickness rotator cuff tears, and bilateral tears are common in clinical practice [1–5]. Previous studies have shown that patients with bilateral rotator cuff tears account for up to 25.9-35.5 % of all rotator cuff tears [1, 4]. A large number of studies have shown significant and predictable clinical benefits of rotator cuff repair in restoring function and reducing pain [6–8]. Patients with bilateral rotator cuff tears are prone to undergo unilateral shoulder surgery to satisfy the demands of daily life [2, 9–11]. There is a current lack of evidence to guide clinical practice on the suitability of bilateral rotator cuff surgery and it is often left to individual preferences in daily clinical practice. Aleem et al. [12] have pointed out that patients who received a staged bilateral rotator cuff repair could obtain similarly good clinical outcomes in both shoulders. Besides, Pak et al. [13] have inferred that single-stage bilateral rotator cuff repair is a good option in selected patients, providing a safe and effective procedure that does not compromise functional results. Hence this study was conducted to compare the postoperative outcomes of the single-stage group and the two-stage group to aid clinicians in guiding patients to make informed decisions. We hypothesized that the two groups would have similar outcomes and both options are comparable.

## Methods

From March 2013 to May 2018, qualified patients were diagnosed to have bilateral rotator cuff tears with symptoms according to preoperative physical examination and radiologic evaluation including magnetic resonance imaging (MRI). The physical examination, including Jobe test, Hawkins test, Neer test and ROM test were performed by the same doctor. The surgical indications were shoulder pain with or without abduction weakness, and patients have undergone failure of conservative management. Inclusion criteria: Patients with bilateral full thickness tears underwent single-stage or staged bilateral rotator cuff repair. Exclusion criteria: Fracture history and previous shoulder surgeries; Other shoulder injuries that would need to be repaired at the time of surgery, such as symptomatic biceps tendinitis or glenoid labrum tears; Patient’s data that were lost during the follow-up.

Pain in bilateral shoulders restrained patients from normal daily life, and they agreed to undergo single-stage or staged bilateral arthroscopic rotator cuff repair according to their own preference. As for patients receiving staged bilateral cuff repair, their more symptomatic shoulder would be repaired first. However, if symptoms of both sides were similar, the dominant shoulder would receive repair first. Basic demographic information of all patients, including age, gender, body mass index (BMI), and tear size, was collected. Before the operation, MRI was used to evaluate the tear size of rotator cuff in all patients. Oblique coronal and oblique sagittal images were obtained with a 3.0-T MRI unit (Siemens Medical Solutions, Erlangen, Germany). The tear size was measured along the anterior-posterior (AP) and medial-lateral (ML) length [14]. At 6, 12, and 24 months postoperatively, routine postoperative MRI was performed. Muscle atrophy and fatty degeneration were evaluated with MRI performed before the operation and at 24 months postoperatively. MRI has comparable accuracy in measuring the tear size of rotator cuff tears. Similarly, it has high accuracy for the detection of rotator cuff healing [15–18]. Muscle atrophy was assessed on oblique sagittal images using the occupation ratio as previously described [19]. Fatty degeneration was assessed according to a 5-point grading system as described by Goutallier et al. [20].

### Operative technique

All patients underwent single-stage or staged rotator cuff repair, and operations were performed by the senior surgeon with 23 years of experience. Patients were placed in the lateral decubitus position when receiving single-stage or staged bilateral cuff repair under general anesthesia. To protect the first operated side in a single-stage procedure, it was well padded in abduction after the patient was turned for the contralateral rotator cuff repair. The posterior portal was established as the viewing portal, and an arthroscope was inserted through this portal to assess intra-articular lesions. Subsequently, the arthroscope was inserted into the subacromial space to view the subacromial lesions and rotator cuff tear. If the patient had any evidence of impingement in subacromial or outlet position, subacromial decompression and acromioplasty was performed to create a type-I flat acromion. Inflamed bursal tissues and adhesions were removed, and the edge of the torn cuff was debrided. The surface of bone was prepared to enhance bone-to-tendon healing. The rotator cuff repair was performed with the single-row technique including suture anchors (Smith & Nephew, USA). Suture anchors were used depend on the size and configuration of the tear. The average time between 1st and 2nd operation was 6 month in the staged group.

### Rehabilitation

After the operation, shoulders were immobilized with an abduction brace for at least 6 weeks. All patients were provided a standard postoperative rotator cuff rehabilitation scheme, which should be strictly obeyed. Passive movement was permitted on the second day after surgery, and active movement was permitted after 6 weeks to gradually increase muscle strength and range of motion (ROM). After the operation, patients were prescribed Celecoxib (200 mg once a day) for 2 weeks.

### Clinical assessment

A visual analog scale (VAS) was used to determine preoperative and postoperative pain. To evaluate clinical outcomes of shoulders before and after the operation, 4 scales were used as follows: University of California, Los Angeles (UCLA) score, consisting of pain(10), function (10), active forward flexion activity (5), strength (5), and patient satisfaction (5); American Shoulder and Elbow Surgeons (ASES), consisting of pain(36), stability(36), and function(28); Constant-Murley score (Constant), consisting of pain(15), activity level(20), active movement(40) and muscle strength(25). The questionnaires were given at 6months, 12 months and 24 months. Patients filled one scale for each shoulder, and average the results. .Active ROM included forward flexion, external rotation at the side, and internal rotation of the shoulder. Active ROM included forward flexion and external rotation at the side measure with a goniometer, and internal rotation measured using the vertebral level reached with the tip of the thumb. The vertebral level was serially scored in this study: 1 point added for each level above the sacrum, with 0 for any level below the sacral region.

### Statistical analysis

SPSS 26.0 statistical software was used for analysis. Data were expressed as mean ± standard deviation (SD). Categorical variables were analyzed using the Chi-square test. An independent sample t-test was adopted to compare quantitative data between groups. ANOVA was employed to compare clinical scores between groups at different time points. The difference between preoperative and postoperative clinical scores of each group was detected using a paired t-test. P < 0.05 was considered statistically significant.

## Results

Of all patients, 51 patients were finally included in this retrospective study, and they attended a minimum clinical follow-up of 24 months. According to the classification criteria proposed by Cofield[21], there were 22 cases of a small tear (0–1 cm), 69 cases of a medium tear (1–3 cm), and 11 cases of a large tear (3–5 cm). Patients were divided into 2 groups. There were 24 patients aged from 39 to 62 (53.8 ± 5.3) years in group A, who underwent single-stage bilateral arthroscopic rotator cuff repair. There were 27 patients aged from 37 to 62 (52.2 ± 6.5) years in group B, who received staged bilateral arthroscopic rotator cuff repair. All incisions healed without any complications. There were no significant differences in terms of the demographic characteristics between the single-stage group and the staged group. (Table 1)

**Table 1:** Demographic data

	Single-stage	Staged	*P* value
Male/female	11/13	12/15	0.842
Age (year)	53.8 (39–62; ±5.3)	52.2 (37–62: ±6.5)	0.353
BMI (kg/m2)	22.1 (18.7–25.4; ±2.8)	22.6 (18.2–26.1: ±2.6)	0.501
Diabetes	3	5	0.838
Smoking history	4	7	0.422
Symptom duration at presentation (month)	6.8 (3–18; ±3.5)	7.6 (5–18; ±3.1)	0.441
Night pain	19	21	0.574
Tear size: small/medium/large	12/31/5	10/38/6	0.729
Isolated supraspinatus	36	39	0.751
supraspinatus + subscapularis	7	8	0.974
supraspinatus + Infraspinatus	5	7	0.690
Atrophy(supraspinatus)	1.60 ± 0.32	1.54 ± 0.31	0.486
Fatty degeneration(supraspinatus)	0.83 ± 0.64	0.87 ± 0.53	0.822

**Fig. 1:**
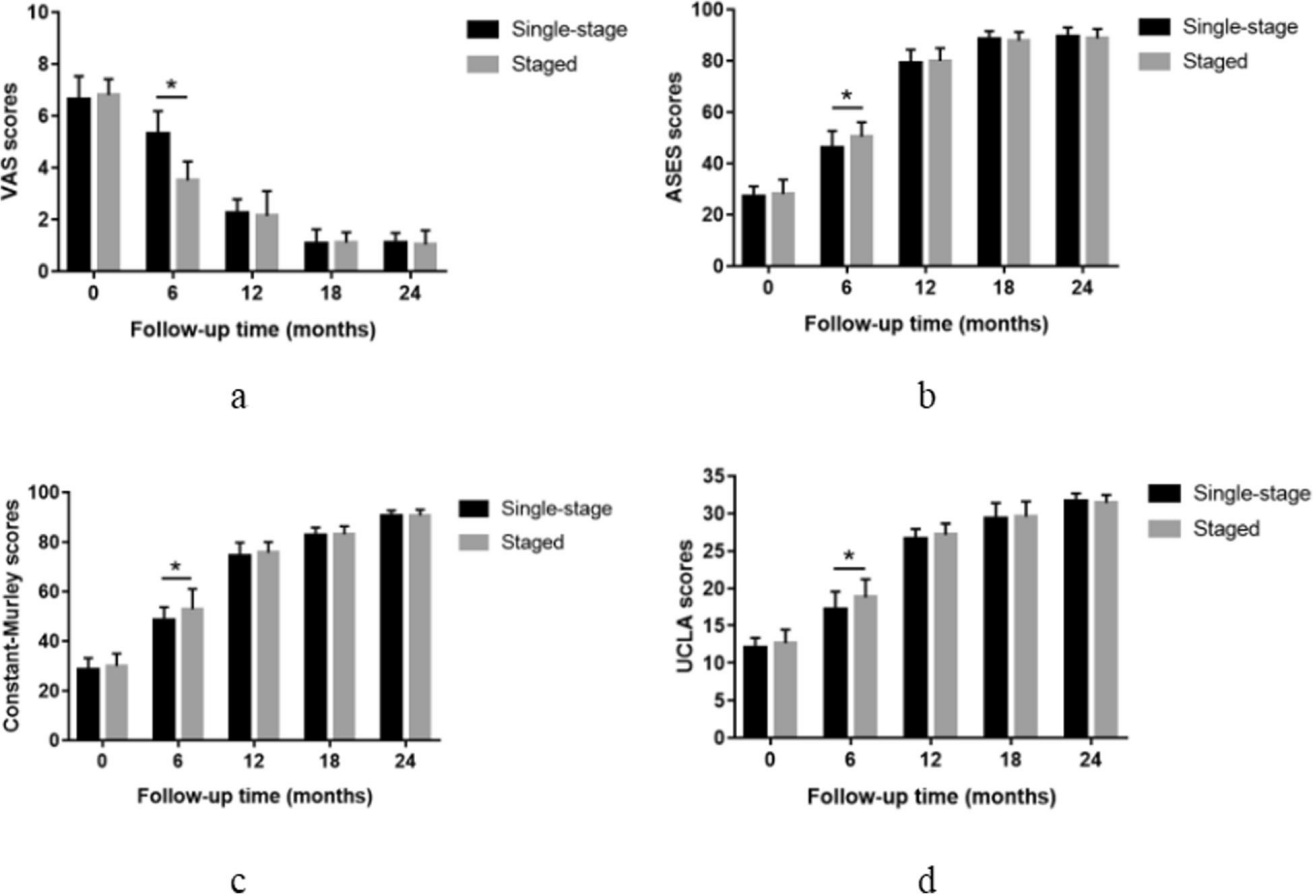
VAS (**a**), ASES (**b**), Constant (**c**), and UCLA (**d**) scores preoperatively and at 6, 12, 18, and 24 months after single-stage vs. staged bilateral rotator cuff repair. *Significant difference (*P* < 0.05)

**Fig. 2:**
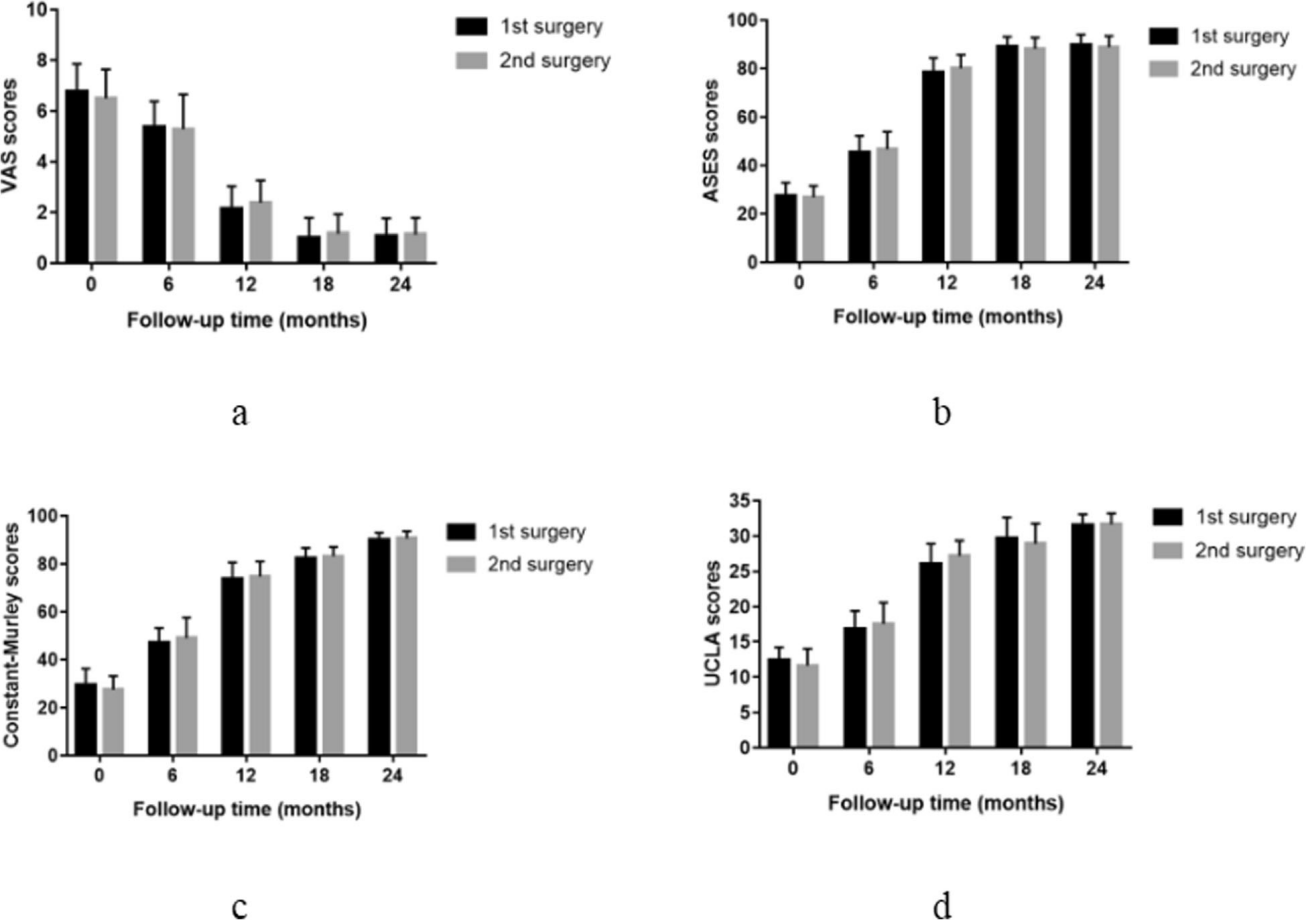
VAS (**a**), ASES (**b**), Constant (**c**), and UCLA (**d**) scores preoperatively and at 6, 12, 18, and 24 months after the first side vs. second side in the single-stage group. No statistically significant differences were found

There was no significant difference in the VAS score (*P* = 0.424), ASES score (*P* = 0.325), UCLA score (*P* = 0.170), and Constant score (*P* = 0.275) between the single-stage group and the staged group before the operation. Compared with the preoperative values in the two groups, VAS score, UCLA score, Constant score, and ASES score were significantly improved at any time point after the operation. The VAS score(5.3 ± 0.89), ASES score(46.04 ± 6.67), UCLA score(17.21 ± 2.35), and Constant score(48.42 ± 5.25) in group A were significantly different from the VAS score(3.5 ± 0.74), ASES score(50.46 ± 5.59), UCLA score(18.78 ± 2.45), and Constant score(52.89 ± 8.13) in group B at 6 months postoperatively (*P* < 0.05). At 12, 18, and 24 months after the operation, there was no significant difference in VAS score, UCLA score, Constant score, and ASES score between the two groups (Fig. 1). Besides, there was no significant difference in VAS score (*P* = 0.295), ASES score (*P* = 0.621), UCLA score (P = 0.248) and Constant score (P = 0.283) between both shoulders in the single-stage group before the operation. Moreover, there was no significant difference in VAS score, UCLA score, Constant score, and ASES score between the first side and second side in the single-stage group postoperatively at follow-up (Fig. 2).

**Fig. 3:**
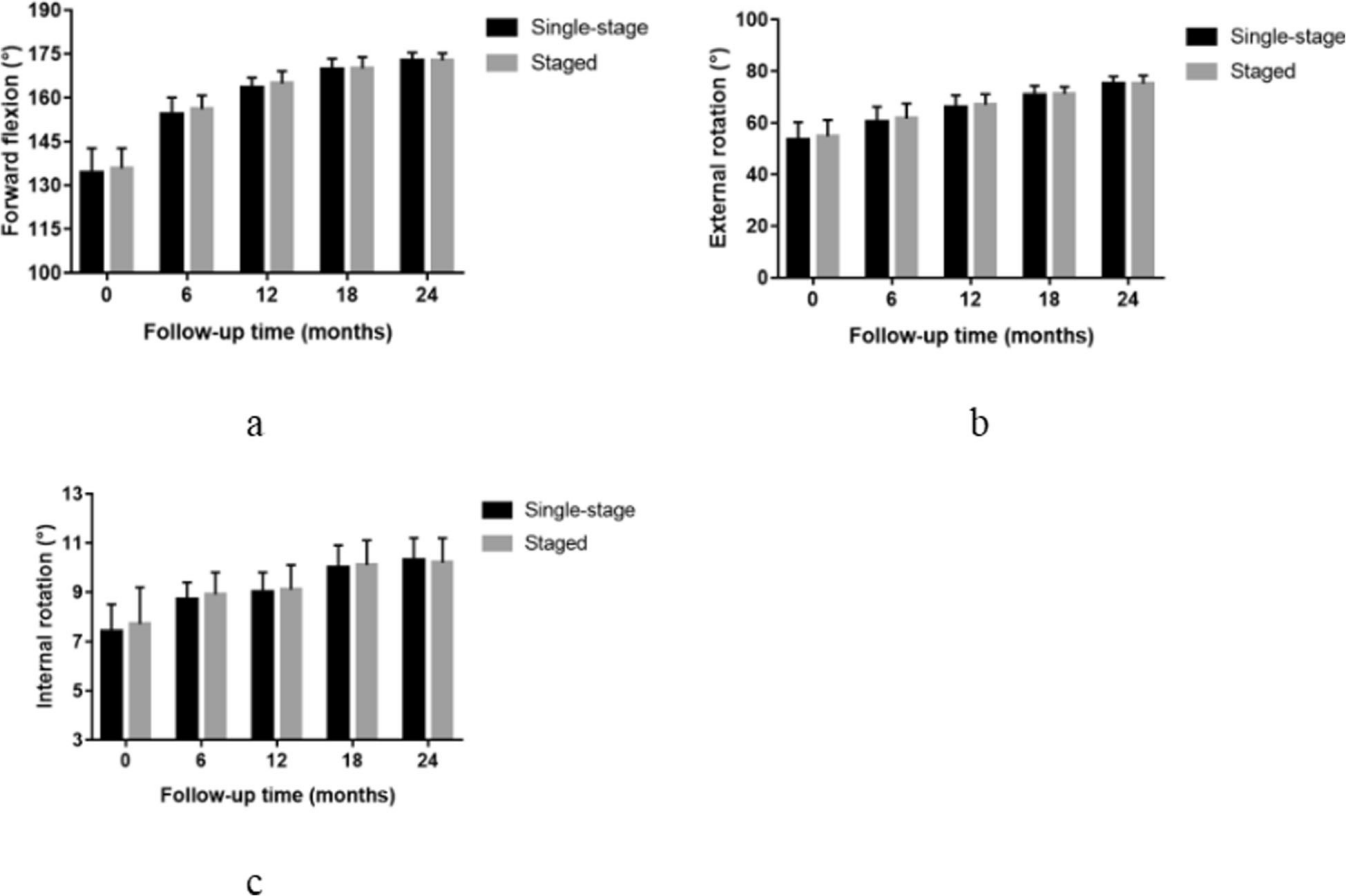
Forward flexion (**a**), external rotation (**b**), internal rotation (**c**) preoperatively and at 6, 12, 18, and 24 months after single-stage vs. staged bilateral rotator cuff repair. No statistically significant differences were found

**Fig. 4:**
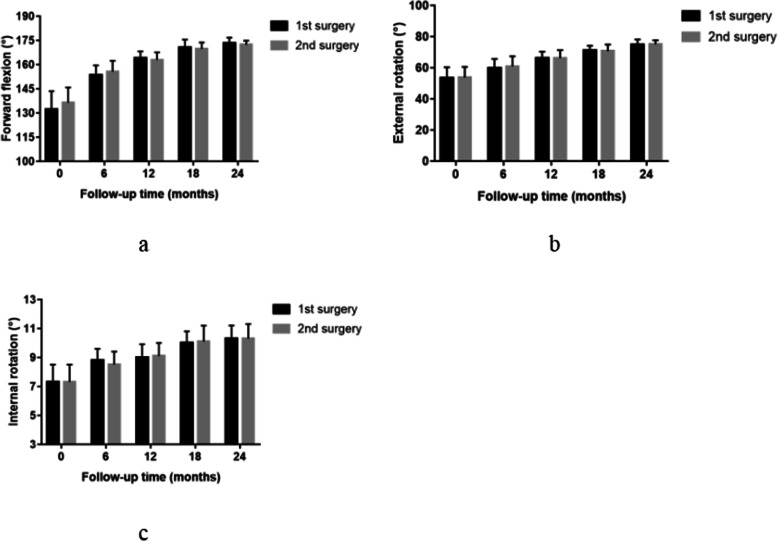
Forward flexion (**a**), external rotation (**b**), internal rotation (**c**) preoperatively and at 6, 12, 18, and 24 months after the first side vs. second side in the single-stage group. No statistically significant differences were found

There was no significant difference in the forward flexion (P = 0.478), external rotation (P = 0.464) at the side, and internal rotation (P = 0.438) of the shoulder between the single-stage group and the staged group before the operation. Compared with the preoperative values in the two groups, the ROM of the shoulder was significantly improved at any time point after operation. At follow-up, the ROM of the shoulder was not significantly different between the two groups (Fig. 3). Besides, there was no significant difference in the forward flexion (P = 0.105), external rotation (P = 0.247) at the side, and internal rotation (P = 0.137) of the shoulder between the first shoulder and second shoulder in the single-stage group before the operation. There was no significant difference in the ROM between the first surgery and second surgery in the single-stage group postoperatively at any time point after operation (Fig. 4).

However, one patient in the single-stage group complained of discomfort of left shoulder after operation. He felt mildly stiff at the beginning of the external rotation of the shoulder. But this symptom disappeared at 6 months after operation.

Postoperative integrity and healing status of the repaired tendon showed no re-tear or other complications in MRI. Table 1 summarizes the MRI findings of muscle atrophy and fatty degeneration between the single-stage group and the staged group before operation, and there was no significant difference in the muscle atroph (*P* = 0.486) or fatty degeneration (*P* = 0.822) between the two groups.

**Fig. 5:**
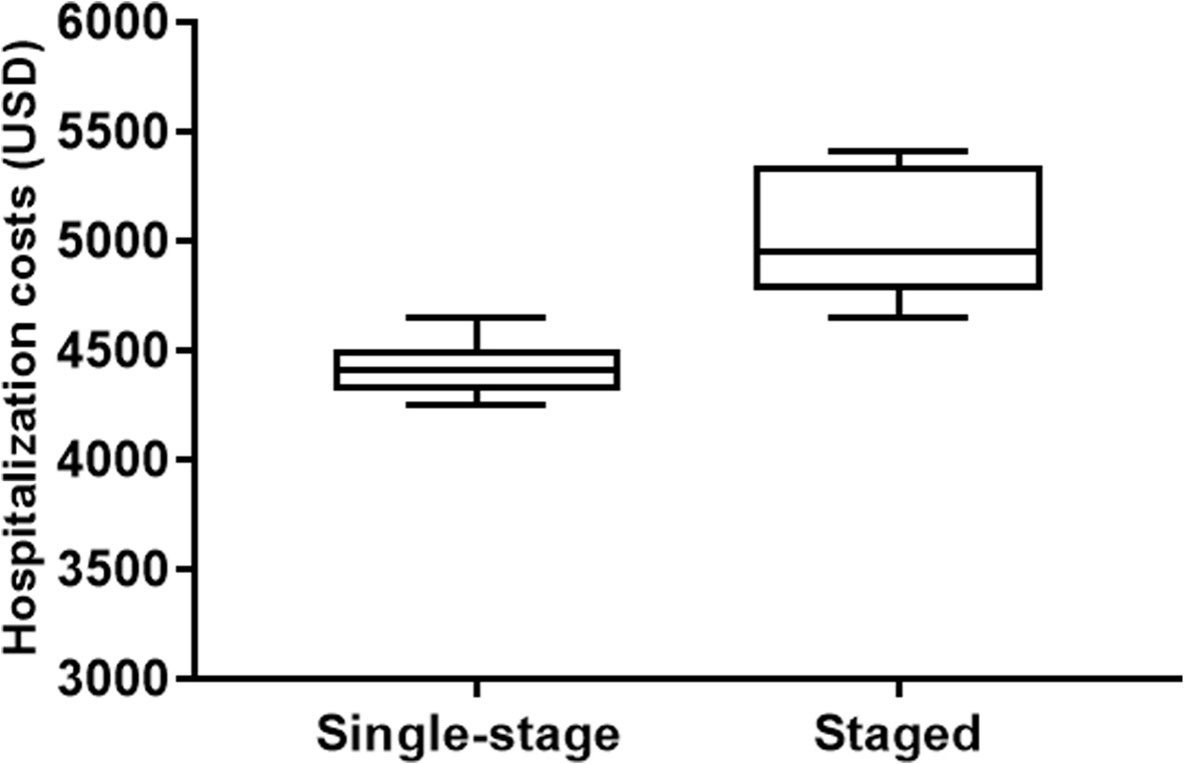
Hospitalization costs in single-stage vs. staged bilateral rotator cuff repair

The average hospitalization costs of patients in the single-stage group and staged group were 4440.89 ± 130.55 and 5065.73 ± 254.76 USD, respectively, and this difference was significant (*P* < 0.001). Box analysis showed that the hospitalization costs of patients in the single-stage group were relatively lower. Besides, the distribution of hospitalization costs in the staged group was relatively scattered. In contrast, the distribution of hospitalization costs in the single-stage group was relatively concentrated(Fig. 5).

## Discussion

In the present study, we compared the outcomes of two surgical timings which would have similar outcomes at the follow-up after the operation. From the results, although the ROM of the shoulder showed similar outcomes between the 2 groups, there were significant difference in function scores and hospitalization costs. The single-stage group showed poorer clinical outcomes of VAS, ASES, UCLA, ASES and Constant scores at 6 months postoperatively, and hospitalization costs were higher in staged group. One patient in the single-stage group felt stiff at the beginning of the external rotation. We considered that excision of synovial tissue, decompression of soft tissue, and long-term immobilization might result in scarring or iatrogenic injury, and the new scar could affect shoulder activity [22, 23], so the post-operative stiffness may be affected by bilateral procedure.

Liem et al. [24] have pointed out that the prevalence of contralateral supraspinatus tears are significantly higher in the surgery group (67.3 %). These findings suggest that patients with rotator cuff tears undergoing surgery have a higher risk of developing a rotator cuff tear on the contralateral side. The probability of bilateral rotator cuff tears is likely to happen while one side tear occurs. Nowadays, patients are prone to accept staged surgery, and single-stage surgery remains uncommon. Therefore, we focused on the outcomes of the two surgical methods. For patients with bilateral rotator cuff tears undergoing staged surgery, a large number of studies have shown that the final result of the second operation is equivalent to the first operation. Rhee et al. [2] have reported that patients with bilateral rotator cuff tears underwent staged surgery. Compared with the first side, the VAS pain score in the second side was significantly worse at 6 months postoperatively. However, there was no significant difference in the VAS score between the two groups at the final follow-up. When all these clinical outcomes from the final follow-up were combined, both sides of shoulders undergoing staged bilateral arthroscopic rotator cuff repairs would get a similarly good result. Patients also accepted staged surgery in our study, the results of the staged group are similar to above studies. A large number of studies have investigated the impact of staged surgery on both shoulders. However, we compared single-stage surgery and staged surgery, and observed the results of postoperative functional scores. From our results in studies, single-stage surgery could be a good substitute with similar clinical outcomes at the final follow-up.

Gerber et al. [25] have demonstrated that single-stage bilateral total joint arthroplasty is considered an alternative to staged bilateral surgery. Compared with the staged group, the postoperative outcomes of the single-stage group are significantly improved, and there are no extra complications in the single-stage group. Although our study was also a comparison between single-stage and staged surgery, protocols may be different because of different surgical methods. For patients who underwent rotator cuff repair, shoulders were immobilized with an abduction brace for at least 6 weeks, but earlier active movement was suggested in above article. Similarly, Pak et al. [13] have focused on the results of a single-stage bilateral rotator cuff repair, including 10 patients receiving single-stage bilateral surgery and 17 patients receiving unilateral surgery. The single-stage bilateral repair offered similar results with no additional complications. They suggest that for patients who can tolerate both shoulder fixation, single-stage bilateral arthroscopic rotator cuff repair is a viable option. In our study, we also investigated the clinical outcomes of single-stage surgery. But we set different groups: single-stage group and staged group. In clinical practice, patients with bilateral rotator cuff tears required bilateral surgery, we intuitively analyzed and compared the two surgical timings. While we also got similar results at final follow-up in the single-stage group, and pointed out the differences between single-stage and staged surgery. Our study was a further investigation for single-stage surgery and provided important clinical guidance for patients with bilateral rotator cuff tears when choosing the appropriate surgical method.

The VAS and functional scores are effective methods to assess postoperative clinical outcomes [26–30]. In our study, at 6 months postoperatively, VAS pain and functional scores after the single-staged operation were worse compared with the staged operation. However, there was no significant difference in VAS pain and functional scores between the two groups at the 12-month follow-up. Because the bilateral rotator cuff tears were repaired at the same time during the single-stage operation, this likely means that patients who accepted single-stage bilateral rotator cuff repair would undergo a more painful experience and more difficult functional rehabilitation. The early postoperative pain and restrictions may be the main reason the single-stage procedure is not routinely performed. Both surgical methods can achieve good functional rehabilitation postoperatively, and there was no difference at the final follow-up. After the two operations, the ROM was significantly improved during the follow-up, and the two surgical methods achieved similarly good results. The repaired tendons were intact, and no obvious postoperative complications were observed in our study.

Due to strict restrictions on both shoulders after the single-stage surgery, they could not perform daily life or early rehabilitation activities [13]. In clinical practice, although patients undergoing single-stage repair needed help with shoulder pad replacement and passive exercise, and some patients slept uncomfortably throughout the night in the early postoperative period, they could adapt and overcome difficulties gradually. We considered that letting patients know the advantages and disadvantages of different surgical options by preoperative conversation was important [31]. Patients in single-stage group have been prepared mentally for difficult postoperative rehabilitation, and one-time recovery was their expectation. Similarly, Patients in staged group were satisfied of easier rehabilitation at the expense of the second surgery. Compared with the staged operation, patients receiving single-stage operation saved hospitalization costs and avoided the second operation. If patients can adapt to the early difficult rehabilitation period and did not require much daily work, we would advise that patients with bilateral rotator cuff tears could consider single-stage bilateral rotator cuff repair. Compared with the staged bilateral rotator cuff repair, good results can also be achieved.

Furthermore, our operation used a lateral position for a single-stage operation. It is generally believed that although the first operation side is protected by thick gauze and shoulder pad, it would be compressed to some extent when patients receiving surgery on the contralateral side [14]. Currently, no research has reported whether the compression of the first operated side in single-stage rotator cuff respire has an impact on postoperative pain, functional scores, and ROM. Our study compared the postoperative outcomes of bilateral shoulders in the single-stage operation. During the follow-up, there was no significant difference in pain scores, functional scores, and ROM. It showed that a short period of intraoperative compression would not affect postoperative functional rehabilitation. Under good protection and short-time operation, short-term compression might not have adverse effects on the first operated side. However, surgeons should thrive to decrease the operative time under the premise of preventing adverse events. A longer operation time would increase the operation risk.

This study provided important clinical guidance for patients who required bilateral rotator cuff repair by the comprehensive comparison between single-stage and staged surgery. This study has several limitations. Because of the strict inclusion criteria, the study population is small and a comparison between the 2 groups may be limited by a possibility of a type 2 error. However, we have to acknowledge that this group of patients are rare and not so common. Besides, this is a retrospective study with all its associated bias, and a prospective randomized controlled studies need to be conducted in the future. The results of this study need to be validated by large clinical samples and prospective randomized controlled studies.

## Conclusions

All 51 patients with bilateral rotator cuff tears were assessed for 2 years after rotator cuff repair. The comparison of clinical scores and MRI showed that both single-staged and staged repair achieved good clinical scores during 2 years of follow-up. There was a small difference in favor of staged surgery at 6 months, but this difference disappeared on subsequent follow-up. Additionally, both the first operation side and the second operation side had good clinical outcomes in the single-stage group. Therefore, single-stage surgery is a good option for patients with bilateral rotator cuff tears, leading to decreased hospitalization and rehabilitation time.

## Data Availability

All data generated or analysed during this study are included in this article.

## Abbreviations

VAS: Visual analog scale
ASES: American Shoulder and Elbow Surgeons score
UCLA: University of California, Los Angeles score
ROM: Range of motion
SD: Standard Deviation
MRI: Magnetic resonance imaging
BMI: Body mass index
AP: Anterior-posterior
ML: Medial-lateral
